# Legacy of pre‐eruption vegetation affects ground‐dwelling arthropod communities after different types of volcanic disturbance

**DOI:** 10.1002/ece3.7755

**Published:** 2021-06-04

**Authors:** Kyohei Iida, Daisuke Hayasaka, Yuya Suzuki, Taizo Uchida, Takuo Sawahata, Koya Hashimoto

**Affiliations:** ^1^ Graduate School of Agriculture Kindai University Nara Japan; ^2^ Kyushu Branch Regional Environmental Planning Inc. Fukuoka Japan; ^3^ Faculty of Agriculture Kindai University Nara Japan; ^4^ Graduate School of Life and Environmental Science University of Tsukuba Tsukuba Japan; ^5^ The United Graduate School of Agricultural Sciences Kagoshima University Korimoto Japan; ^6^ Faculty of Architecture and Civil Engineering Kyushu Sangyo University Fukuoka Japan; ^7^ National Institute for Environmental Studies (NIES) Tsukuba Japan

**Keywords:** community resilience, disturbance ecology, ecological succession, large natural disturbance, legacy effects, volcanic island

## Abstract

Volcanic eruptions are one of the largest natural disturbances and are followed by the establishment of novel plant and animal communities in terrestrial ecosystems. However, the role of pre‐eruption vegetation in the establishment of arthropod communities after volcanic disturbances is currently unknown. Here, we asked whether the legacy of pre‐eruption vegetation mediates the community structure of ground‐dwelling arthropods after volcanic disturbances. The 2015 eruption in Kuchinoerabu‐jima Island, southwest Japan, caused two types of disturbances [a pyroclastic flow and a lahar (i.e., mudflow)] in three types of forests (broad‐leaved, black pine, and cedar). We hypothesized that pre‐eruption vegetation would influence the community structure of ground‐dwelling arthropods after the disturbance, and we expected that these effects from vegetation would be more prevalent for the less severe disturbances. The total abundance of ground‐dwelling arthropods decreased more in the lahar than the pyroclastic flow, and arthropod species composition showed a greater change after the lahar. These findings suggest that the lahar disturbance was more severe than the pyroclastic disturbance. Contrary to expectations, the difference in the arthropod species composition among the vegetation types was greatest after the lahar. After the pyroclastic flow, leaf litter remained to some degree with all the vegetation types. After the lahar disturbance, however, although the litter in the cedar forests remained, the litter disappeared completely from broad‐leaved and black pine forests. The disappearance of litter from these two forest types after the lahar may be responsible for the greater difference in arthropod species composition among the vegetation types. This study shows that the legacy effects of pre‐eruption vegetation on terrestrial arthropod communities after volcanic disturbance were different depending on the type of disturbance. Focusing on the role of pre‐eruption biotic factors would contribute to a better understanding of the recovery processes of terrestrial ecosystems after large natural disturbances.

## Graphical abstract

Our paper found that the legacy of pre‐eruption vegetation influenced the community structure of ground‐dwelling arthropods after volcanic disturbances. Moreover, counterintuitively, these legacy effects of vegetation were more evident for the more severe disturbances. This figure shows how biological legacy of forests remains after receiving the volcanic disturbances.

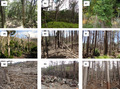

## 1 INTRODUCTION

Natural disturbances are one of the ecological processes that contribute to the establishment and maintenance of biodiversity and ecosystems (Schowalter, 2012). Disturbances such as hurricanes, volcanic eruptions, fires, earthquakes, tsunamis, and population explosions of consumer species such as pests vary in their frequency, intensity, and magnitude (Barbosa et al., 2012; Berenstecher et al., 2017; Hayasaka, Goka et al., 2012; Hayasaka, Shimada et al., 2012; He et al., 2019; Spiller & Agrawal, 2003). Depending on the type of disturbance, the responses of biotic organisms to these events are expected to differ greatly (Walker & Willing, 1999; White & Pickett, 1985), leading the structure of communities and ecosystems to various alternative states including the recovery of predisturbance conditions (del Moral & Magússon, 2014). Thus, variations in the organismal responses are critical to understand the resilience of communities and ecosystems against natural disturbances (e.g., Dale, et al., 2005; Hayasaka, et al., 2016; Ishida, et al., 2015). Recently, several studies have indicated that biological legacies, which are defined as biotic factors such as residual organisms and physical structures that persist in an ecosystem following a disturbance, are an important but largely unrecognized determinant of postdisturbance ecosystem resilience (Bergeron et al., 2017; Johnstone et al., 2016).

Among natural disturbances, volcanic eruptions are one of the largest in terms of magnitude and intensity and can cause dramatic changes in terrestrial and aquatic ecosystems (del Moral & Grishin, 1999). Although studies on the responses of terrestrial ecosystems to volcanic eruptions have largely focused on the succession of plant communities to date (Berenstecher et al., 2017; del Moral & Magússen, 2014; Kondo et al., 2016; Tsuyuzaki, 2009; Walker & del Moral, 2003), there is evidence that insects and other arthropods are vulnerable to volcanic eruptions as well (Elizalde, 2014; Schowalter, 2012). The physical impact force exerted by pyroclastic flows (i.e., rushing currents generated by volcanic eruptions) and subsequent burial, together with the lethal effects of volcanic ash, can cause extinctions and population declines of broad arthropod taxa, resulting in the loss of species richness (Sikes & Slowik, 2010). On the other hand, volcanic disturbances may facilitate colonization by novel arthropods into an environment newly created by these disturbances. In fact, after large eruptions, ground‐dwelling arthropods such as ants, spiders, woodlice, and beetles reportedly colonized and established their populations on the disturbed ground earlier than did pioneer plant species (Edwards, 1986; Edwards & Sugg, 2005; Edwards & Thornton, 2001; New, 2008), probably facilitating the succession of terrestrial ecosystems (Edwards, 1986; Edwards & Sugg, 2005). Ground‐dwelling arthropods have diverse ecosystem functions, including decomposing organic matter, transferring energy by predation, acting as vectors of seed dispersal, and improving the soil quality (Hölldobler & Wilson, 1990; Lal, 1988; Seastedt & Crossley, 1984); consequently, revealing the characteristics of ground‐dwelling arthropod communities established after volcanic disturbances may help to clarify how volcanism determines the successional transitions of terrestrial communities. However, because of their infrequent nature, the impacts of volcanic eruptions on ecological communities are difficult to investigate (Berenstecher et al., 2017), and thus, the ecological consequences of the legacies of pre‐eruption biotic factors for ecosystem recovery are unknown.

The establishment of ground‐dwelling arthropod communities after volcanic disturbances may be influenced by the legacy of pre‐eruption biotic factors such as vegetation types. The legacy effects of vegetation on posteruption arthropod communities may arise from at least two avenues. First, because terrestrial arthropod communities tend to differ depending on the vegetation structure (Lövei & Sunderland, 1996), posteruption arthropod communities consisting of surviving individuals would also differ among pre‐eruption vegetation structures. Second, the legacy effects of pre‐eruption vegetation may occur through colonization by arthropods from surrounding areas (c.f., Bengtsson et al., 2003). Because the physical structure specific to each vegetation type may remain to some degree even after large disturbances (Johnstone et al., 2016; Sasal et al., 2010), the type of vegetation that was present prior to the eruption would mediate posteruption physical environments such as habitat openness and litter deposition. This variation in environments due to pre‐eruption vegetation types would influence the colonization and establishment of communities with different species richness levels and compositions.

Moreover, the legacy effects of pre‐eruption vegetation may depend on the type of volcanic disturbance. A volcanic eruption triggers various types of disturbances including lavas, ash falls, pyroclastic flows, and lahars (i.e., mudflows caused by quakes due to volcanic eruption), and these disturbance types often have different intensities in terms of their ecological impact (del Moral & Grishin, 1999). In particular, lavas, pyroclastic flows, and lahars are generally intensive disturbances, such that they often initiate primary succession (del Moral & Grishin, 1999). Given that intensive disturbances would decrease the survival probabilities of arthropods and that the physical structures of vegetation are less likely to remain unchanged upon receiving more intensive disturbances, any legacy effects of vegetation on arthropod communities are expected to become weaker when a more intensive type of disturbance occurs. However, there are no studies examining whether and how the types of volcanic disturbances determine or influence the legacy effects of pre‐eruption vegetation on the arthropod community structure.

The large volcanic eruption that occurred on May 29, 2015, at Kuchinoerabu‐jima Island, which is an active volcanic island in southwestern Japan and has diverse temperate–subtropical vegetation types, offers an opportunity to examine the interactive effects of pre‐eruption vegetation types and different types of volcanic disturbances on terrestrial communities. This eruption caused various types of disturbances including a pyroclastic flow, a lahar, ash falls, and hot gasses. Among these, the pyroclastic flow and the lahar were the most conspicuous disturbances of this eruption. However, there were also undisturbed areas near the affected places, which served as reference areas. At each location receiving one of the above three disturbance conditions (i.e., undisturbed, the pyroclastic flow, and the lahar), there were existing vegetation structures of evergreen broad‐leaved forests, black pine forests, and cedar forests, enabling a comparison of effects after the eruption.

In this study, we aimed to examine whether the legacy of pre‐eruption vegetation mediates the community structure of ground‐dwelling arthropods after the volcanic eruption disturbances on Kuchinoerabu‐jima Island in relation to the types of volcanic disturbance. For this purpose, we conducted a field survey to monitor arthropod communities at sites with different combinations of three disturbance conditions, namely undisturbed, the pyroclastic flow, and the lahar, and three vegetation types: evergreen broad‐leaved forest, black pine forest, and cedar forest. Since the vegetation structure plays a critical role in shaping the associated terrestrial arthropod communities (Lövei & Sunderland, 1996), we hypothesized that the species composition of ground‐dwelling arthropods in the undisturbed places would differ among the vegetation types. We also expected that the arthropod assemblages would become more similar after a more severe disturbance because the homogenization of physical environments may facilitate a recolonization by arthropods with similar habitat preferences and/or because only more disturbance‐tolerate species may survive after severe disturbances (c.f., Sasal et al., 2010). In addition, we measured two physical environmental parameters (light intensity, which was considered as a proxy of habitat openness, and litter depth) at each monitoring site to infer the mechanisms underlying the effects of volcanic disturbances and vegetation types on arthropod communities. These mechanisms would influence the colonization and/or establishment of arthropod communities after disturbances.

Specifically, we addressed the following questions. (1) Does the community structure of ground‐dwelling arthropods differ, after a volcanic disturbance, depending on pre‐eruption vegetation types? (2) If so, does the prevalence of legacy effects relating to pre‐eruption vegetation types differ between the two types of volcanic disturbances (i.e., the pyroclastic flow and the lahar)? (3) What factors are responsible for and mediate the legacy effects of pre‐eruption vegetation on arthropod communities?

## 2 MATERIALS AND METHODS

### 2.1 Study area and description of the eruption

This study was conducted in late October both 2016 and 2017 at the Mukaehama and Maeda areas, in a cove of mid‐west Kuchinoerabu‐jima Island (30°28′N, 130°12′E, Figure S1). Kuchinoerabu‐jima is an active volcanic island located about 12 km northwest from Yakushima Island, Kagoshima Prefecture, southern Japan. This island has several volcanos including Mt. Shindake, which has erupted repeatedly during the modern period, with an interval of about 20 years (Tameguri et al., 2016). Mt. Shindake erupted on the morning of 29 May 2015, resulting in severe disturbances to the ecosystem of Kuchinoerabu‐jima Island. This eruption was relatively large and generated two types of volcanic disturbance. A pyroclastic flow generated by this explosive eruption moved from the main crater toward the west‐northwest and reached Mukaehama beach (AIST, 2015). The temperature of the gas of this pyroclastic flow was 100–400 ℃, which was relatively low compared to the average (AIST, 2015). Also, this pyroclastic flow consisted mainly of hot gas and ashes, but the deposition of ashes caused by this flow was not large (up to 1 cm). On 3 June 2015, a lahar, which occurred due to repeated rains after the day of the eruption, moved toward Mukaehama beach (Kyushu Regional Development Bureau & MLIT, 2015). The depth of the deposition of mud, debris, and boulders (some of which had diameters above 1 m) from the lahar is estimated to be up to 2 m or more (the first author, personal observation). The distance between the edge (i.e., adjacent to undisturbed area) and the middle of the disturbed area (which was disturbed by the pyroclastic flow and/or the lahar) was approximately 400 meters at a maximum, in which recolonization by arthropods surviving in undisturbed areas into the disturbed areas may not have been difficult.

**FIGURE 1:**
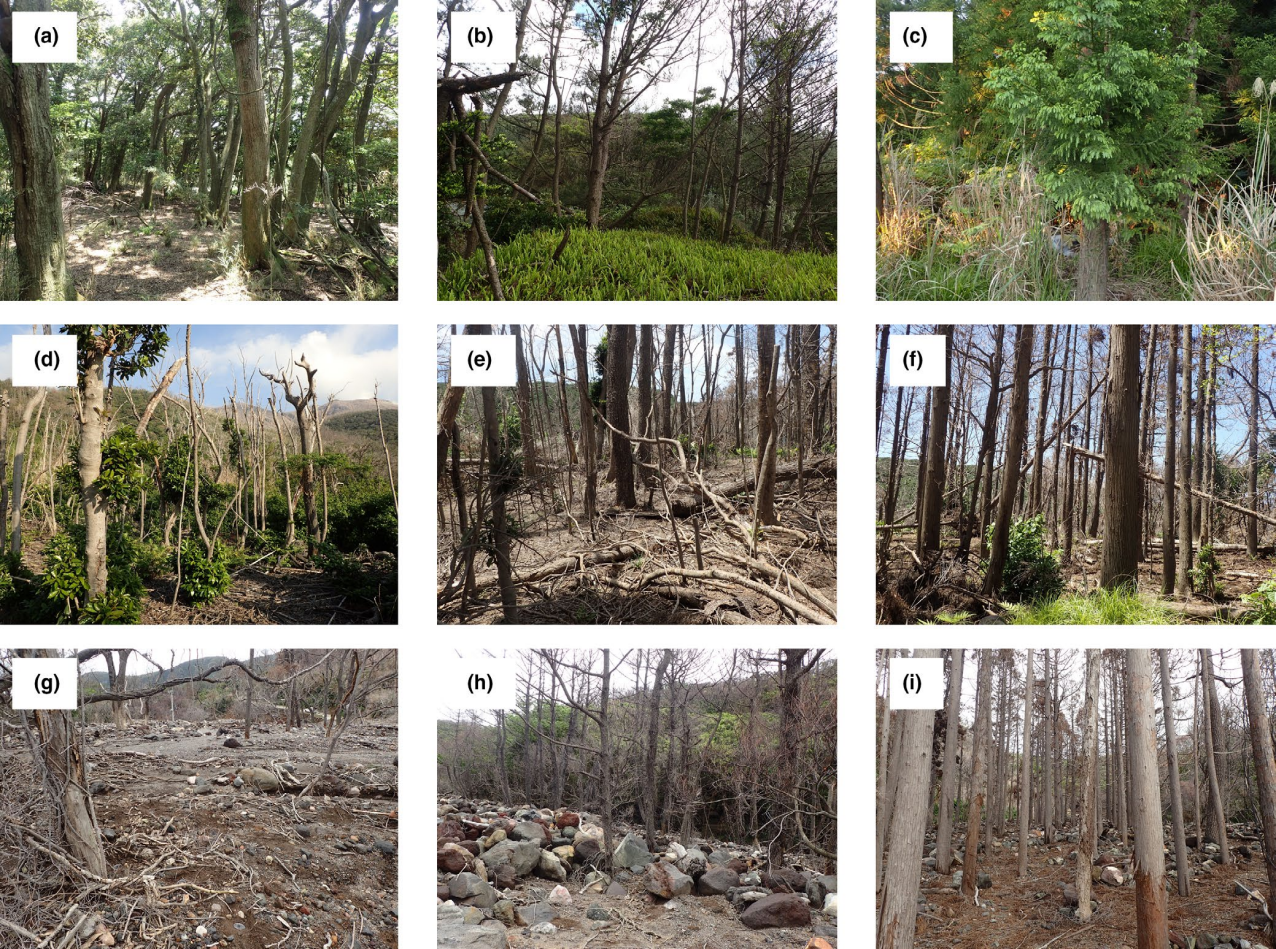
Each study environment. (a) Broad‐leaved, undisturbed forest (BU), (b) black pine, undisturbed forest (PU), (c) cedar, undisturbed forest (CU), (d) broad‐leaved forest in the pyroclastic flow (BP), (e) black pine forest in the pyroclastic flow (PP), (f) cedar forest in the pyroclastic flow (CP), (g) broad‐leaved forest in the lahar (BL), (h) black pine forest in the lahar (PL), and (i) cedar forest in the lahar (CL)

The vegetation of the Mukaehama and Maeda areas consisted primarily of three types of forest stands: evergreen broad‐leaved forest mainly consisting of *Oreocnide pedunculata* and *Trema orientalis*, black pine (*Pinus thunbergii*) forest, and cedar (*Cryptomeria japonica*) forest (GIS data, Biodiversity Center of Japan; http://gis.biodic.go.jp/webgis/sc‐002.html#webgis/453051). Pictures of forests receiving the volcanic disturbances are given in Figure 1. Forests of all vegetation types in this island broadly experienced one of the two types of volcanic disturbance (i.e., the pyroclastic flow or the lahar), but the three vegetation types remained undisturbed in some places of the Mukaehama and Maeda areas; thus, we had 3 × 3 = 9 combinations of disturbance condition and forest type (Figure 1). In the pyroclastic flow, almost all the trees died while standing, except for some individual broad‐leaved trees that survived. The forest floor vegetation started to recover within 2–3 years in these places. In the lahar, most broad‐leaved trees and black pines were knocked down and buried by lahar deposits. A few trees were not knocked down but died while standing. By contrast, most cedars were not knocked down but died while standing with withered leaves on their canopies. The ground vegetation did not show any recovery even 3 years after the eruption.

### 2.2 Field survey

To examine whether the community structures of ground‐dwelling arthropods differed depending on the conditions of the volcanic disturbance and the vegetation type, we surveyed ground‐dwelling arthropod communities in the Mukaehama area after the volcanic disturbance. By using pitfall traps, we monitored arthropod communities at nine types of sampling sites, which were the full combinations of the three conditions of volcanic disturbance (i.e., undisturbed, the pyroclastic flow, and the lahar) and the three vegetation types (broad‐leaved forest, black pine forest, and cedar forest). We arbitrarily selected five points in each combination as sampling sites, comprising 45 points in total (Figure S1). The minimum, mean, and maximum distances between the points were 27.3 m, 306.9 m, and 895.8 m, respectively. The monitoring was conducted two times on 18–20 October 2016, and during a year later on 27–29 October 2017 (Table S1). The timing of this sampling falls within the typical range of the phenology during which arthropods are active in Japan. At each sampling site, we buried 10 plastic cups (8 cm diameter × 7 cm depth) at an interval of 1 m. The cups were filled with surfactant‐solvated water to a depth of approximately 2 cm without baits, and 24 hr later, we collected all individuals of ground‐dwelling arthropods from these cups. The collected arthropods were preserved in 70% ethanol and were brought to our laboratory. We identified all arthropods by species or morphospecies within known genera or families using a dissecting microscope (SMZ1500, Nikon Instech, Tokyo, Japan).

Along with the biotic monitoring, we also assessed two environmental parameters: relative light intensity and deposited litter depth. We measured the light intensity at each sampling site at 10–11 a.m. in 2017 using illuminant meters (LX‐1108, SATOTECH, Kanagawa, Japan). We simultaneously measured the light intensity in an open area next to each sampling site as a control and calculated the relative light intensity (%) as (light intensity at a sampling site / control light intensity) × 100. The depth of the litterfall was measured by inserting a measuring device into the litter layer.

### 2.3 Statistical analyses

All statistical analyses were performed using R statistical software version 3.4.1 (R Core Team, 2017). To determine whether and how the conditions of disturbance impacted arthropod community properties and whether the legacy of vegetation types differentiated arthropod community properties after a disturbance, we performed (generalized) linear mixed modeling analyses. We focused on three community properties: total abundance, species richness, and species evenness. Because the number of species sampled is expected to depend on the sampling effort, we used rarefied species richness rather than the raw data of the number of species by individual‐based rarefaction procedure (Gotelli & Colwell, 2001). Specifically, based on Hurlbert's (1971) theoretical formulation, we rarefied the number of species in all the samples down to the total abundance in the sample with the lowest abundance (Crutsinger et al., 2008). Rarefaction was performed by the function “rarefy” in the package “vegan” version 2.4.6 (Oksanen et al., 2018). As an index of species evenness, we calculated the commonly used Pielou's evenness *J*’. The *J*’ value ranged from 0 to 1. The higher the *J*’ value is, the higher the evenness in the community. For each community property, we constructed a mixed model containing disturbance condition (undisturbed, pyroclastic flow, or lahar) and vegetation type (broad‐leaved forest, black pine forest, or cedar forest) and their interaction as explanatory variables. Site identity and year were included as random effects. We assumed a negative binomial error distribution for total abundance and normal error distributions in relation to species richness and evenness. When assuming a normal error distribution, the data were ln(*x*)‐transformed to meet the assumption of normality and homoscedasticity when necessary. The significance of each term was tested using type III likelihood ratio tests for the negative binomial model and type III *F* tests with Kenward–Roger degrees of freedom for the normal models, followed by Tukey tests among different disturbance conditions and different vegetation types within each disturbance condition. Note that rarefied richness and evenness may be elevated in the disturbed sites only because the abundant taxa in the undisturbed sites were decreased by disturbances. In addition, the presence of one colony of colonial species (i.e., Formicidae) could cause the capture of hundreds or thousands of individuals, perhaps leading to distorted results. Therefore, we performed additional analyses for the above community properties in which Formicidae, which was the most abundant family (see Results), was omitted from the data to confirm whether this family compromised the results (especially for rarefied richness and evenness). The negative binomial and normal models were constructed using the packages “glmmTMB” version 1.0.2.1 (Brooks et al., 2017) and “lme4” version 1.1.14 (Bates et al., 2017), respectively.

To visualize whether and how disturbance conditions and vegetation types altered the species compositions of ground‐dwelling arthropods, we performed nonmetric multidimensional scaling (NMDS) with Bray–Curtis dissimilarity. The data were log(*x* + 1)‐transformed before calculating the Bray–Curtis dissimilarity. To obtain biplots of taxa and sites from the NMDS, we overlaid vectors representing correlations between the counts of each family at each site and the site scores along the NMDS ordination axes (Legendre & Gallagher, 2001) using the function “envfit” in the package “vegan” version 2.4.6 (Oksanen et al., 2018). Only statistically significant correlations were displayed. Then, we performed a permutation MANOVA (PERMANOVA) with 4,999 permutations based on the dissimilarity calculated above to examine the effects of disturbance condition and vegetation type on species composition. The explanatory variables were disturbance condition, vegetation type, and year and their interactions. Since our survey design involved repeated measures but was not balanced (i.e., disturbance ×vegetation × year combinations had unequal numbers), we also performed another PERMANOVA with restricted permutation to account for repeated measures while excluding the sites that lacked data. Because the results were not qualitatively different between the two PERMANOVAs, we reported the results of the former analysis. Since there was a possibility of spatial autocorrelation, we further performed an additional analysis considering spatial autocorrelation. The analysis was performed in two steps. First, we constructed linear models by each census year with spherical spatial correlations using the function “gls” in the package “nlme” version 3.1.131 (Pinheiro et al., 2017). The response variables were the values of NMDS axis 1, but we did not include any explanatory variables other than intercepts. Next, we extracted the residuals from these models and constructed a linear mixed model including the residuals as response variables and disturbance condition, vegetation type, and their interactions as explanatory variables, and year and site identity were included as random effects. This analysis can be interpreted to examine the effects of the disturbance condition and vegetation type on species composition after statistically removing spatial autocorrelations. Furthermore, to understand the consequences of arthropod composition changes due to disturbances for future ecosystem succession, we employed an analysis examining whether and how the distribution of the feeding guilds (i.e., omnivores, carnivores, detritivores, herbivores, and scavengers) of the arthropods differed depending on disturbance conditions and vegetation types. We constructed a linear mixed model in which the log(*x* + 1)‐transformed abundance of each feeding guild as a response variable and disturbance condition, vegetation type, feeding guild, and their interactions were explanatory variables. Year and site identity were included as random effects. A normal error distribution was chosen because any generalized methods (i.e., linear models assuming non‐normal error distributions) failed to fit our data well. When the disturbance × vegetation × guild interaction was significant (as examined by *F* test with Kenward–Roger degrees of freedom), we judged that the composition of the feeding guilds differed among vegetation types and that these differences depended on disturbance condition. Additionally, we constructed similar models for each disturbance condition separately and then tested the significance of the vegetation × guild interaction by *F* tests to examine if the composition of feeding guilds differed among vegetation types for different disturbance conditions.

Environmental data were analyzed by ANOVAs to examine the effects of disturbance condition and vegetation type on relative light intensity and litter deposition. Relative light intensity was arcsine square‐root‐transformed, and litter deposition was square‐root‐transformed to meet the assumption of normality and homoscedasticity. Tukey tests among different vegetation types in each disturbance condition followed each ANOVA.

## 3 RESULTS

### 3.1 Description of arthropod samples

In total, we obtained 14,719 individuals of ground arthropods. We identified 129 species or morphospecies from 51 families of 14 orders (see Table S2, for full descriptions of the observed species or morphospecies). At the family level, the ground‐dwelling arthropods consisted primarily of Formicidae (ants, 80.2%), followed by Porcellionidae (woodlice, 4.8%), Lycosidae (wolf spiders, 3.3%), Gryllidae (crickets, 3.2%), Staphylinidae (rove beetles, 2.6%), Linyphiidae (sheet weaver spiders, 1.3%), and Carabidae (ground beetles, 0.85%).

### 3.2 Community properties of ground‐dwelling arthropods

**FIGURE 2:**
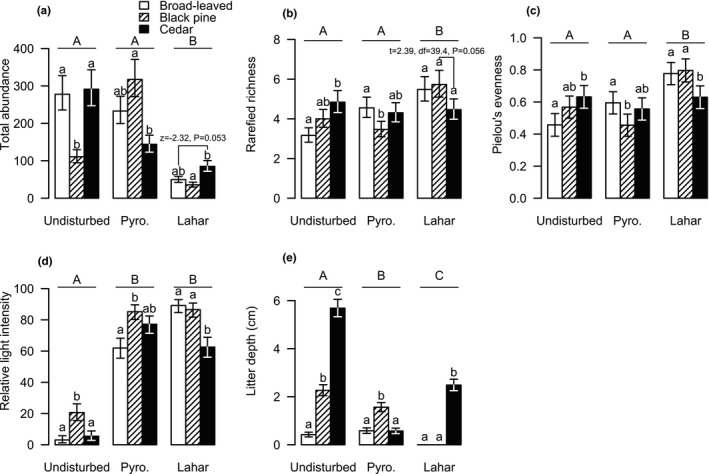
Differences in the (a) total abundance, (b) rarefied richness, and (c) Pielou's evenness of ground‐dwelling arthropods, (d) relative light intensity, and (e) litter depth among the three vegetation types (broad‐leaved forest, black pine forest, and cedar forest) under each disturbance condition (undisturbed, in the pyroclastic flow, and in the lahar) following the 2015 eruption of Shin‐take, Kuchinoerabu‐jima. Back‐transformed least square means ± *SE* are shown. Different uppercase and lowercase letters indicate statistical significance among the disturbance conditions and the vegetation types within a disturbance condition, respectively

The lahar decreased the total abundances of ground‐dwelling arthropod communities more than did the pyroclastic flow (Figure 2a). The total abundance of ground arthropods at the sites in the lahar was significantly lower than that at the undisturbed sites, while that at the sites in the pyroclastic flow did not differ from that at the undisturbed sites (Figure 2a). Vegetation type altered the total abundance of ground arthropods, depending on the disturbance conditions (Table 1). In particular, cedar forest sites had the highest total abundances among the undisturbed sites and the sites in the lahar, but they had the lowest total abundance among the sites in the pyroclastic flow (Figure 2a). Moreover, a separate pairwise comparison showed that black pine forest sites in the pyroclastic flow had higher total abundance relative to undisturbed black pine forest sites (*Z* = −4.76, *p* < .001).

**TABLE 1:** The effects of the volcanic disturbance condition and vegetation legacy on arthropod community metrices

Community properties	Explanatory variables	LR‐*χ* ^2^ */F*	*df*	*p*
Total abundance	Disturbance condition (D)	65.61	2	**<.001**
	Vegetation legacy (V)	7.60	2	.**02**
	D × V	30.60	4	**<.001**
Rarefied richness	Disturbance condition (D)	13.31	2,34.9	**<.001**
	Vegetation legacy (V)	0.58	2,35.0	.57
	D × V	8.02	4,34.9	**<.001**
Pielou's evenness	Disturbance condition (D)	23.39	2,34.9	**<.001**
	Vegetation legacy (V)	0.007	2,35.0	.99
	D × V	6.79	4,34.9	**<.001**

Significant values indicated in bold.

In contrast to total abundance, the rarefied richness and evenness (*J’*) of ground‐dwelling arthropods were not diminished but were even increased slightly by the volcanic disturbances, although species density (i.e., the nonrarefied number of species) was slightly lowered at the lahar sites (Figure S2). In particular, the sites in the lahar showed relatively higher rarefied richness and evenness levels of ground arthropod communities compared to undisturbed sites and those affected by the pyroclastic flow (Figure 2b, c). Moreover, the vegetation type altered the species richness and evenness of ground arthropods, depending on the conditions of the disturbance (Table 1). The rarefied richness of ground arthropods at cedar forest sites was the highest among the undisturbed sites, while it was intermediate among the sites in the pyroclastic flow and the lowest among the sites in the lahar (Figure 2b). The *J’* values of ground arthropods showed almost the same pattern as that of the rarefied richness (Figure 2c). Overall, broad‐leaved forest and black pine forest sites in the lahar showed the highest rarefied richness and evenness in ground arthropods among all the surveyed sites (Figure 2b, c). Note that when we removed Formicidae from our analyses, which was the most abundant family in our study, the legacy effects of vegetation on the rarefied richness and evenness were no longer observed, but there was still a tendency of rarefied richness and evenness to not be diminished by the volcanic disturbances (Figure S3).

### 3.3 Species composition of ground‐dwelling arthropods

**FIGURE 3:**
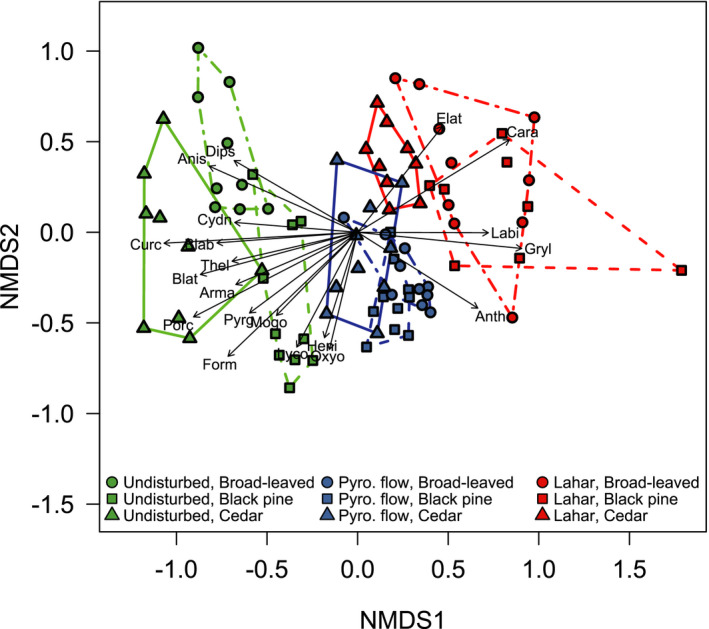
Nonmetric multidimensional scaling (NMDS) plot of the ground‐dwelling arthropod species compositions after the 2015 eruption of Shin‐take, Kuchinoerabu‐jima. Green, blue, and red points represent the undisturbed communities, those in the pyroclastic flow, and those in the lahar. Circles, squares, and triangles represent the communities in broad‐leaved, black pine, and cedar forests. Stress value = 0.18. The vectors describe changes in the abundance of each family. The family abbreviations are as follows: Dips, Dipsocoridae; Anis, Anisolabididae; Cydn, Cydnidae; Curc, Curculionidae; Blab, Blaberidae; Thel, Thelyphonidae; Blat, Blattellidae; Arma, Armadillidae; Porc, Porcellionidae; Pyrg, Pyrgodesmidae; Form, Formicidae; Mogo, Mogoplistidae; Lyco, Lycosidae; Heni, Henicopidae; Oxyo, Oxyopidae; Elat, Elateridae; Cara, Carabidae; Labi, Labiduridae; Gryl, Gryllidae; and Anth, Anthicidae

The NMDS ordination showed that the variation of the species composition among the surveyed sites was primarily explained by the conditions of the volcanic disturbance (Figure 3; PERMANOVA, disturbance; *F*_2,67_ = 29.34, *p* < .001). The ground‐dwelling arthropod communities of undisturbed sites and the sites in the pyroclastic flow and the lahar were located along NMDS axis 1 from left to right in this order. The vegetation type also influenced the species composition (Figure 3; PERMANOVA, vegetation type; *F*_2,67_ = 8.54, *p* < .001), and its effects depended on the conditions of the volcanic disturbance (PERMANOVA, disturbance × vegetation; *F*_4,67_ = 5.82, *p* < .001). The species composition at undisturbed sites was located at different positions in the multivariate species space among different vegetation types, while the compositions at the sites in the pyroclastic flow that had different vegetation types were not clearly separated from each other compared to that at undisturbed sites (Figure 3). However, for the sites in the lahar, which was the more severe disturbance, the species composition at the cedar forest sites was separated clearly from that at broad‐leaved and black pine forest sites and was located near those of the sites in the pyroclastic flow (Figure 3). Such disturbance‐dependent effects of vegetation on the species composition were evident even after removing the effects of potential spatial autocorrelations (Figure S4). When we removed spatial autocorrelations, disturbance ×vegetation interaction was significant (*F*_4,35.5_ = 3.8, *p* < .05). Pairwise comparison revealed that there were no significant differences in the residuals among vegetation types at the sites in the pyroclastic flow but that the cedar forest sites in the lahar were significantly different relative to the broad‐leaved and pine forest sites in the lahar (Figure S4). These results indicate that the effects of the pre‐eruption vegetation on the arthropod species composition were more prevalent after the lahar than the pyroclastic flow.

We identified 20 families showing statistically significant correlations between the counts of each family and the NMDS site scores. Along NMDS axis 1, which may reflect the severity of the volcanic disturbance, 15 families (Dipsocoridae, Anisolabididae, Cydnidae, Curculionidae, Blaberidae, Thelyphonidae, Blattellidae, Armadillidae, Porcellionidae, Pyrgodesmidae, Formicidae, Mogoplistidae, Lycosidae, Henicopidae, and Oxyopidae) decreased in their abundance (Figure S5), while the abundances of 5 families (Elateridae, Carabidae, Labiduridae, Gryllidae, and Anthicidae) increased (Figure S6). Moreover, these families increased along NMDS axis 1 and did not show high abundances at cedar forest sites in the lahar, except for Gryllidae (Figure S6).

The distribution of arthropod feeding guilds changed depending on the disturbance condition and vegetation type interactions (Figure S7; disturbance × vegetation × guild interaction; *F*_16,344.0_ = 8.63, *p* < .001). The abundance rank of each guild was omnivores, carnivores, detritivores, herbivores, and scavengers in general. However, detritivore abundance was high at the undisturbed cedar sites, suggesting the effects of vegetation type on the feeding guild composition within the undisturbed sites (Figure S7; vegetation × guild interaction; *F*_8,112.2_ = 17.72, *p* < .001). In contrast with the undisturbed sites, within the sites in the pyroclastic flow, the distribution of feeding guilds no longer significantly differed among vegetation types (Figure S7; vegetation × guild interaction; *F*_8,122.0_ = 1.88, *p* = .069). Furthermore, within the sites in the lahar, the statistically significant effect of vegetation type on feeding guild composition was also suggested, but *F* statistics suggested that the significance of the effect was weaker than that within the undisturbed sites (Figure S7; vegetation ×guild interaction; *F*_8,107.6_ = 2.85, *p* < .01). Specifically, detritivores, herbivores, and scavengers almost disappeared from the broad‐leaved and black pine forest sites, whereas they were still present at cedar forest sites (Figure S7).

### 3.4 Physical environments

Volcanic disturbances significantly influenced relative light intensity (*F*_2,36_ = 142.36, *p* < .001) and litter depth (*F*_2,36_ = 128.55, *p* < .001). Relative light intensities at the sites in the pyroclastic flow and lahar were considerably higher than that at undisturbed sites (Figure 2d). Although we found significant effects of vegetation type on relative light intensity (*F*_2,36_ = 8.51, *p* < .001), which depended on volcanic disturbance (disturbance ×vegetation; *F*_2,36_ = 4.17, *p* < .01), the effects were not as obvious as the effects of disturbance (Figure 2d). Unlike light intensity, litter depth largely differed among different vegetation types (*F*_2,36_ = 160.27, *p* < .001), depending on disturbance condition (disturbance ×vegetation interaction; *F*_2,36_ = 64.34, *p* < .001; Figure 2e). Among the undisturbed sites, cedar forest sites showed deeper litter than did broad‐leaved forest and black pine forest sites (Figure 2e). For the sites in the pyroclastic flow, the litter depth of the cedar forest sites considerably decreased to the level of broad‐leaved and black pine forests. However, when the lahar occurred, the litter of cedar forest sites maintained its depth, whereas litter completely disappeared from the broad‐leaved and black pine forest sites.

## 4 DISCUSSION

This is the first study demonstrating how the types of volcanic disturbances and vegetation legacy interactively influence the community structure of ground‐dwelling arthropods. The legacy effects of vegetation on the compositions of the ground‐dwelling arthropod communities were evident after both the pyroclastic flow and the lahar (Figure 3). Moreover, the legacy effects of vegetation on arthropod species composition were more prevalent for the lahar than for the pyroclastic flow (Figure 3), even though in this study, the lahar disturbance had greater effects on the total abundance and species composition of arthropods than did the pyroclastic flow disturbance (Figures 2 and 3). Within the lahar, the arthropod species composition of the cedar forest sites was clearly different from those of the broad‐leaved forest and black pine forest sites. In addition, the litter deposition results suggest that litter, which serves as food and/or shelter for ground‐dwelling arthropods, remained only in the cedar forests among the lahar sites. These results indicated that the legacy effects of vegetation on ground‐dwelling arthropod communities after volcanic disturbances were altered by the different types of disturbances and that this alteration was not solely explained by the intensity of the disturbance but also by other factors such as the atypical response of the cedar forests in terms of the litter deposition.

### 4.1 Effects of different types of volcanic disturbances on ground‐dwelling arthropod communities

In general, terrestrial arthropods are negatively affected by volcanic disturbances (Elizalde, 2014). Likewise, in this study, most of the ground‐dwelling arthropod taxa apparently experienced negative effects from the volcanic eruption. Along NMDS axis 1, which may reflect in part the intensity of the disturbance, 15 families including Formicidae, Porcellionidae, Lycosidae, and Curculionidae decreased their abundance, as mentioned above (Figures 3 and S5). The negative effects of the volcanic disturbances may have been derived from direct impacts and burial by volcanic materials (Edwards & Sugg, 2005; Sikes & Slowik, 2010), the alteration of physical environments (Schowalter, 2012), and the lethal effects of volcanic ash (Edwards & Schwaltz, 1981). Among these, burial may have been the major mechanism responsible for the remarkable declines in the total abundance of arthropods due to the lahar, as shown by the large deposits of volcanic materials, mud, and debris of up to two meters (Figure 1). Similar burial results were reported for Mount St. Helens, where a deep debris avalanche preceded by a pyroclastic blast buried arthropods and decreased their abundance dramatically (Edwards & Sugg, 2005). In addition to this direct effect of burial, deposits from the lahar may have buried litter in broad‐leaved and black pine forests, resulting in the alteration of physical habitats for arthropods. Most ground‐dwelling arthropods are known to use leaf litter as food and/or habitat resources (Bastow, 2011; Santos et al., 1978). Thus, the disappearance of litter may have negatively affected those arthropods that survived burial by the lahar.

Unlike most arthropod taxa, we found five families (Elateridae, Carabidae, Labiduridae, Gryllidae, and Anthicidae) which increased in terms of their abundance along NMDS axis 1 (Figures 3 and S6). In particular, this was true for all the species of Carabidae and Gryllidae, as well as *Labidura riparia* (Labiduridae) and *Agrypnus miyamotoi* (Elateridae), which are generalist carnivores and/or scavengers. In general, arthropods that initially colonize bare ground and become dominant are not primary consumers but rather secondary or higher order consumers (Hodkinson et al., 2002), because carnivores can prey on animals dispersed by wind, and scavengers can feed on the carcasses of other animals, even on bare ground (Crawford et al., 1995; Edwards & Sugg, 2005; Hodkinson et al., 2002). In fact, previous studies reported that arthropod carnivores and scavengers became dominant on the bare ground of Mount St. Helens where a pyroclastic flow occurred (Edwards, 1986; Edwards & Sugg, 2005). These feeding guilds can facilitate the colonization of bare ground by other organisms, accelerating nutrient cycling (Edwards & Sugg, 2005; Hodkinson et al., 2002) and subsequently driving successional changes in ecosystems after volcanic disturbances.

In this study, the arthropod species compositions at the sites in the pyroclastic flow were plotted between those of the undisturbed sites and the sites in the lahar on the NMDS ordination (Figure 3). The 15 families that dramatically decreased in terms of their abundance at the sites in the lahar also decreased at the sites in the pyroclastic flow, but some families including the Formicidae and Lycosidae did not show obvious abundance decreases at the sites affected by the pyroclastic flow (Figure S5). For Formicids, in particular, it is likely that individuals below ground (i.e., in their nests) could survive a pyroclastic flow. In addition, the total abundance of arthropods did not clearly decrease due to the pyroclastic flow (compared to that at the undisturbed sites), and at the black pine forest sites, the abundance even increased (Figure 2a). Pyroclastic flows are generally believed to be catastrophic disturbances for arthropod communities (Elizalde, 2014), which is seemingly not consistent with our results. This inconsistency may be due to (1) the relatively low temperature of the pyroclastic flow (AIST, 2015) and (2) the relatively few deposits of pyroclastic materials (up to 1 cm) in this eruption, allowing the soil surface to remain intact. Previous studies suggested that the type of volcanic disturbances affects the impacts of the disturbances on terrestrial communities (Parmenter et al., 2005). In this context, our results emphasized that the effects of the type of volcanic disturbances are not consistent across systems and/or studies and that other factors such as litter depth rather than type itself should be considered to understand the potential ecological impacts of volcanic disturbances.

### 4.2 Legacy effects of pre‐eruption vegetation on ground‐dwelling arthropod communities dependent on the types of volcanic disturbances

Our study clearly showed that the legacy of vegetation types influenced the species compositions of ground‐dwelling arthropods after volcanic disturbances and that these legacy effects were more prevalent at the sites in the lahar compared to the pyroclastic flow (Figure 3). The legacy effects of vegetation on the arthropod species composition may have resulted from various mechanisms. First, differences in the arthropod species composition before disturbances can remain even after disturbances if some of the original arthropods survive. In general, the vegetation structure plays a critical role in shaping the associated terrestrial arthropod communities (Lövei & Sunderland, 1996). In fact, the undisturbed sites on Kuchinoerabu‐jima Island showed different species compositions among the three vegetation structures (broad‐leaved forests, black pine forest, and cedar forests). These differences may have also existed at the sites in the pyroclastic flow or the lahar before the disturbances (although we have no data on the arthropods present before the disturbances), leading to the observed legacy effects of vegetation on the species composition of ground‐dwelling arthropods. Second, the modification of physical environmental parameters by disturbances may have depended on the existing vegetation types, thus resulting in colonization by arthropods with different habitat preferences. In this study, we found that the cedar forest sites in the lahar had an approximately 3‐cm litter depth, while broad‐leaved and black pine forest sites in the lahar had no litter deposits (Figure 2e). Thus, litter deposits in cedar forests may have provided food or habitat resources to some ground‐dwelling arthropods, ameliorating the negative impacts of the lahar on such communities. In a previous study, the effects of natural fires on ground‐dwelling arthropods such as cockroaches and woodlice were weaker at forest sites, where ample amounts of leaf litter existed, than in open lands with scarce litter deposits (Kwok & Eldridge, 2015). Moreover, the lack of litter deposits at broad‐leaved and black pine forest sites may have facilitated colonization by carnivores and scavengers in the families of Carabidae, Labiduridae, and Elateridae, which increased in number with increasing intensities of the disturbances (Figures 3 and S6). Thus, the legacy effects of vegetation on the arthropod species composition at the sites with lahar can be partly explained by colonization by carnivores and/or scavengers at the sites without litter deposits. The results of the rarefied richness, which were the highest in the broad‐leaved and black pine forest sites with the lahar (Figure 2b), also supported our view that these sites may have been colonized by the newly arrived species, although these results may be solely due to the decrease in abundant taxa such as Formicidae (Figure S3).

There are at least two possible reasons why cedar forests retained leaf litter even at the sites affected by the lahar. First, leaves that fell from canopies after the disturbance may have generated the litter deposits. At the sites in the lahar, trees from all the forest types died while standing, and while leaf litter could fall from dead standing trees in any type of forest, cedars are known to produce more litter from their canopies compared to broad‐leaved trees and black pines (Yoshida, 2006). The characteristics of the cedars may explain the thicker litter deposition observed at the cedar forest sites. Second, the decomposition rate of cedar litter may be slower than those of broad‐leaved and black pine forest litter. Nakane (1995) reported that the litter in cedar forests was more slowly decomposed than that of broad‐leaved and pine forests, probably due to the great resistance of cedar litter to decomposition. This property may have resulted in the obviously deeper litter at the cedar forest sites in the undisturbed sites and the sites in the lahar. Third, given that the density of dead standing trees seemed to be higher in the cedar forest than for the other two vegetation types (Figure 1g, h, and i), this higher density may have helped block the wind and thus preserved fallen leaves on the ground, although we did not measure the actual density of dead standing trees in this study. Leaf litter is less likely to be deposited on bare ground under the direct action of wind and/or rain than in dense forests, even when the forests are dead. At less‐dense broad‐leaved and black pine forest sites, the wind and/or rain may have eliminated litter deposits even when additional leaf litter fell from dead standing trees.

Unlike the lahar sites, the species compositions of arthropod communities at the pyroclastic flow sites were similar among the different types of vegetation (Figure 3). This suggests that the arthropod species composition became more similar among the different vegetation types after the pyroclastic flow. Similar responses by arthropods to large disturbances have been reported, for example, following a large forest fire, which made the species compositions of ground‐dwelling arthropods among different vegetation types more similar (Sasal et al., 2010). It is unclear why the legacy effects of vegetation on the species composition of arthropods were less prevalent for the pyroclastic flow compared to the lahar, but there is a possibility that hot gas blowing in the pyroclastic flow eliminated most of the leaves in all the forest types, and thus, litter deposits became thinner for all vegetation types. In fact, the litter depth in the cedar forest at the pyroclastic flow sites was thinner than that in the cedar forests within the lahar sites. Taken together, to understand the legacy effects of vegetation on terrestrial ecosystems following different types of volcanic disturbances, we need to focus not only on the intensity of the disturbances but also on more detailed information about the modifications of the physical environment.

### 4.3 Implications for ecosystem recovery after volcanic disturbances

Volcanic disturbances are one of the largest natural disturbances, and their consequences for communities and ecosystems are diverse and complex. Moreover, since volcanic disturbances are relatively rare events, it is difficult to make generalities about whether and how the ecosystems receiving volcanic disturbances recover their predisturbance states or shift to alternative states. In this context, the effects of biological legacies on disturbed communities and ecosystems have been suggested to be key to understanding ecosystem resilience against natural disturbances, because biological legacies could act as “memories” of predisturbance conditions (Johnstone et al., 2016). In this study, pre‐eruption vegetation types differentiated the community composition of ground‐dwelling arthropods, suggesting the effects of biological legacies of pre‐eruption vegetation on arthropod communities.

Our study suggests that the difference in litter depths among the combinations of disturbance conditions and vegetation types was a key factor. In particular, the presence of litter deposition in disturbed sites may have offered resources such as food and/or shelter for ground‐dwelling arthropods. In fact, the broad‐leaved and black pine forest sites in the lahar, which had no litter deposition, had almost no detritivorous and herbivorous arthropods, whereas at the other sites there were detritivores and herbivores. Given that the cedar forest litter from undisturbed and disturbed sites have the same ecosystem functions, this litter may act as a memory of predisturbance ecosystem conditions, which would facilitate the recovery of terrestrial communities and ecosystems to predisturbance conditions; for example, recolonization of disturbed sites with leaf litter deposition by detritivores may facilitate fast nutrient cycling through the digestion of the litter. Moreover, our study suggests that the consequences of legacy effects for community and ecosystem resilience may be mediated by the types of volcanic disturbances; the pyroclastic flow may have a spatially homogenized community structure of ground‐dwelling arthropods among the sites with different pre‐eruption vegetation, whereas after the lahar, the variation in the arthropod community structure was still present, probably due to the differentially changed physical environment such as the litter depth. To understand the role of biological legacies in community and ecosystem recovery after volcanic disturbances, we need to reveal what factors force the legacy effects of pre‐eruption biotic factors to direct future transitions in disturbed ecosystems toward pre‐eruption states or other alternative states.

## 5 CONCLUSION

This study indicated that (1) the legacy of pre‐eruption vegetation certainly influenced the community structure of ground‐dwelling arthropods and (2) the type of volcanic disturbance differently altered the legacy effects of pre‐eruption vegetation types on the arthropod communities. Previous studies examining the effects of volcanic eruptions on terrestrial arthropods rarely considered the biological legacies of pre‐eruption biotic factors. Moreover, there are few studies examining the community‐level effects of volcanic eruptions on arthropods. In this context, our study emphasizes the need for considering community properties and the legacy of vegetation structures for a more comprehensive understanding of arthropod responses to volcanic eruptions. Given the importance of ground‐dwelling arthropods for ecosystem function (Lal, 1988; Seastedt & Crossley, 1984), these studies can help predict the resilience and future transitions of terrestrial communities after volcanic events. Finally, our findings may be extrapolated to not only other volcanic eruptions but also other types of large natural disturbances such as landslides, forest fires, hurricanes, and tsunamis. Future studies on the ecological consequences of large natural disturbances should move from individual‐ and population‐level approaches to community‐level approaches and incorporate the role of the biotic environment before the disturbances.

## CONFLICT OF INTEREST

We declare no conflict of interest.

## AUTHOR CONTRIBUTION

**Kyohei Iida:** Conceptualization (lead); Data curation (lead); Formal analysis (equal); Investigation (lead); Methodology (equal); Visualization (equal); Writing‐original draft (equal). **Daisuke Hayasaka:** Conceptualization (supporting); Funding acquisition (lead); Project administration (lead); Resources (lead); Supervision (lead); Validation (equal); Writing‐original draft (supporting). **Yuya Suzuki:** Data curation (supporting); Investigation (supporting); Writing‐review & editing (equal). **Taizo Uchida:** Conceptualization (supporting); Writing‐review & editing (equal). **Takuo Sawahata:** Conceptualization (supporting); Funding acquisition (supporting); Project administration (equal); Resources (supporting); Validation (equal); Writing‐review & editing (equal). **Koya Hashimoto:** Conceptualization (supporting); Formal analysis (equal); Methodology (equal); Visualization (equal); Writing‐original draft (equal).

## Supporting information

Appendix S1

## Data Availability

Data available from the Figshare Repository: https://doi.org/10.6084/m9.figshare.14608320
