# Supplementary material for: Legacy of pre‐eruption vegetation affects ground‐dwelling arthropod communities after different types of volcanic disturbance
Source: Ecol Evol. 2021 Jun 4;11(13):9110–22. doi: 10.1002/ece3.7755 (PMC8258224; doi:10.1002/ece3.7755)
Supplement: Supplementary file 1 — Appendix S1 [file ECE3-11-9110-s001.pdf]

Supporting information.

Title

Legacy of pre-eruption vegetation affects ground-dwelling arthropod communities after different types of volcanic disturbance.

Authors

Kyohei Iida†, Daisuke Hayasaka†, Yuya Suzuki, Taizo Uchida, Takuo Sawahata, and Koya Hashimoto*

*: For correspondence (atrophaneura4@gmail.com (K. Hashimoto))

†: Equal contribution

Ecology and Evolution

Table S1. Summary of field survey sampling. The surveyed sites and each combination in each study year are shown. Each open circle indicates that sampling was performed at each site during each year. The location of each study site is shown in Fig. S1.

Forest type	Broad-leaved					Black pine					Cedar				
Disturbance condition	Undisturbed														
Site name	BU1	BU2	BU3	BU4	BU5	PU1	PU2	PU3	PU4	PU5	CU1	CU2	CU3	CU4	CU5
2016		○	○	○	○	○	○	○	○	○	○	○		○	○
2017	○	○	○	○	○	○	○	○	○	○	○	○	○	○	○

Forest type	Broad-leaved					Black pine					Cedar				
Disturbance condition	Pyroclastic flow														
Site name	BP1	BP2	BP3	BP4	BP5	PP1	PP2	PP3	PP4	PP5	CP1	CP2	CP3	CP4	CP5
2016	○	○	○	○	○	○	○	○	○	○	○	○	○	○	○
2017	○	○	○	○	○	○	○	○	○	○	○	○	○	○	○

Forest type	Broad-leaved					Black pine					Cedar				
Disturbance condition	Lahar														
Site name	BL1	BL2	BL3	BL4	BL5	PL1	PL2	PL3	PL4	PL5	CL1	CL2	CL3	CL4	CL5
2016	○	○	○	○	○		○	○	○	○	○	○	○		○
2017	○	○	○	○	○	○		○	○	○	○	○	○	○	○

Table S2. Ground-dwelling arthropod species found in the present study.

Order	Family	(Morpho)Species	Total abundance
Araneae	Araneae	Araneae sp.	2
	Ctenidae	Ctenidae sp.	3
	Ctenizidae	Ctenizidae sp.	1
	Cybaeidae	*Cybaeus* sp.	1
	Eutichuridae	*Cheiracanthium eutittha*	1
	Gnaphosidae	*Zelotes asiaticus*	2
	Hahniidae	*Hahnia thorntoni*	2
	Linyphiidae	*Erigone edentata*	111
		Erigone prominens	19
		Linyphiidae sp. 1	8
		Linyphiidae sp. 2	17
		Linyphiidae sp. 3	15
		Linyphiidae sp. 4	3
		Linyphiidae sp. 5	1
		Linyphiidae sp. 6	4
		Nematogmus sanguinolentus	8
		Walckenaeria sp.	1
	Lycosidae	*Arctosa* sp.	29
		Lycosa coelestis	19
		Lycosa sp.	172
		Pardosa laevitarsis	18
		Pardosa oriens	2
		Pardosa sp.	242
		Tricca japonica	3
		Trochosa aquatica	2
	Miturgidae	*Itaysina praticola*	1
	Nesticidae	*Nesticella mogera*	1
	Oonopidae	*Ischnothyreus narutomii*	4
	Oxyopidae	Oxyopidae sp.	3
	Salticidae	*Evarcha* sp.	1
		Salticidae sp.	3
		Sitticus penicillatus	1
		Synagelides annae	1
	Sparassidae	*Heteropoda* sp.	1
	Tetrablemmidae	*Ablemma shimojanai*	39
	Theridiidae	Theridiidae sp.	2
	Zodariidae	*Asceua japonica*	1
Archaeognatha	Machilidae	*Pedetontus* sp.	41
Blattodea	Blaberidae	*Opisthoplatia orientalis*	5
	Blattellidae	*Blattellidae* sp.	7

Table S2. (continued)

Order	Family	(Morpho)Species	Total abundance
Coleoptera	Anthicidae	*Anthicidae* sp.	4
		Anthicus protensus	1
	Bolboceratida	*Bolbocerosoma nigroplagiatum*	1
	Carabidae	*Bembidion* sp.	115
		Chlaenius posticalis	1
		Cicindela japonica	7
		Harpalus niigatanus	2
	Curculionidae	*Shirahoshizo rufescens*	1
		Syrotelus umbrosus	1
		Trachyrhinus sp.	72
	Elateridae	*Agrypnus miyamotoi*	20
	Scarabaeidae	*Rhyparus azumai*	1
	Silphidae	*Necrodes nigricornis*	1
	Staphylinidae	Scydmaeninae sp.	7
		Staphylinidae sp. 1	2
		Staphylinidae sp. 2	18
		Staphylinidae sp. 3	230
		Staphylinidae sp. 4	25
		Staphylinidae sp. 5	69
		Staphylinidae sp. 6	1
		Staphylinidae sp. 7	3
		Staphylinidae sp. 8	1
		Staphylinidae sp. 9	4
		Staphylinidae sp. 10	1
		Staphylinidae sp. 11	5
		Staphylinidae sp. 12	1
		Staphylinidae sp. 13	2
		Staphylinidae sp. 14	1
		Staphylinidae sp. 15	1
		Staphylinidae sp. 16	4
		Staphylinidae sp. 17	1
Dermaptera	Anisolabididae	*Anisolabella marginalis*	8
	Labiduridae	*Labidura riparia*	32
Hemiptera	Cydnidae	Cydnidae sp.	10
	Dipsocoridae	Dipsocoridae sp.	27
	Lygaeidae	Lygaeidae sp.	2
	Rhyparochromidae	Rhyparochromidae sp.	5
	Saldidae	Saldidae sp.	3
	Schizopteridae	Schizopteridae sp.	1

Table S2. (continued)

Order	Family	(Morpho)Species	Total abundance
Hymenoptera	Bethylidae	Bethylidae sp.	4
	Formicidae	*Camponotus devestivus*	2
		Camponotus nawai	2
		Camponotus nipponicus	1
		Cardiocondyla itsukii	420
		Crematogaster osakensis	50
		Crematogaster vagula	3
		Leptogenys confucii	1
		Monomorium chinense	879
		Monomorium floricola	1
		Myrmecina nipponica	1
		Pachycondyla pilosior	2
		Pachycondylaa chinensis	46
		Paratrechina amia	30
		Paratrechina flavipes	201
		Paratrechina sakurae	17
		Pheidole fervens	6501
		Pheidole noda	2999
		Pheidole pieli	90
		Ponera scabra	3
		Ponera sp.	8
		Pristomyrmex punctatus	4
		Solenopsis japonica	7
		Strumigenys godeffroyi	27
		Technomyrmex brunneus	10
		Tetramorium bicarinatum	275
		Tetramorium lanuginosum	81
		Tetramorium nipponense	15
		Vollenhovia benzzai	123
Isopoda	Armadillidae	Armadillidae sp.	11
	Porcellionidae	*Burmoniscus* sp.	712
Isoptera	Rhinotermitidae	*Reticulitermes speratus*	21
Lithobiomorpha	Henicopidae	Henicopidae sp.	25
Neuroptera	Myrmeleontidae	*Distoleon nigricans*	6
Orthoptera	Acrididae	*Oedaleus infernalis*	1
	Gryllidae	*Lexoblemmus* sp.	271
		Sclerogryllus puctatus	3
		Teleogryllus occipitalis	3
		Teleogryllus sp.	188
		Velarifictorus aspersus	5
		Velarifictorus micado	5
	Mogoplistidae	*Ornebius kanetataki*	6
	Tetrigidae	*Euparatettix insularis*	16
		Tetrigidae sp.	38
	Trigonidiidae	*Dianemobius nigrofasciatus*	20
		Polionemobius mikado	64
		Pteronemobius nigrescens	1
Polydesmida	Pyrgodesmidae	Pyrgodesmidae sp. 1	16
		Pyrgodesmidae sp. 2	1
Thelyphonida	Thelyphonidae	*Typopeltis stimpsonii*	15

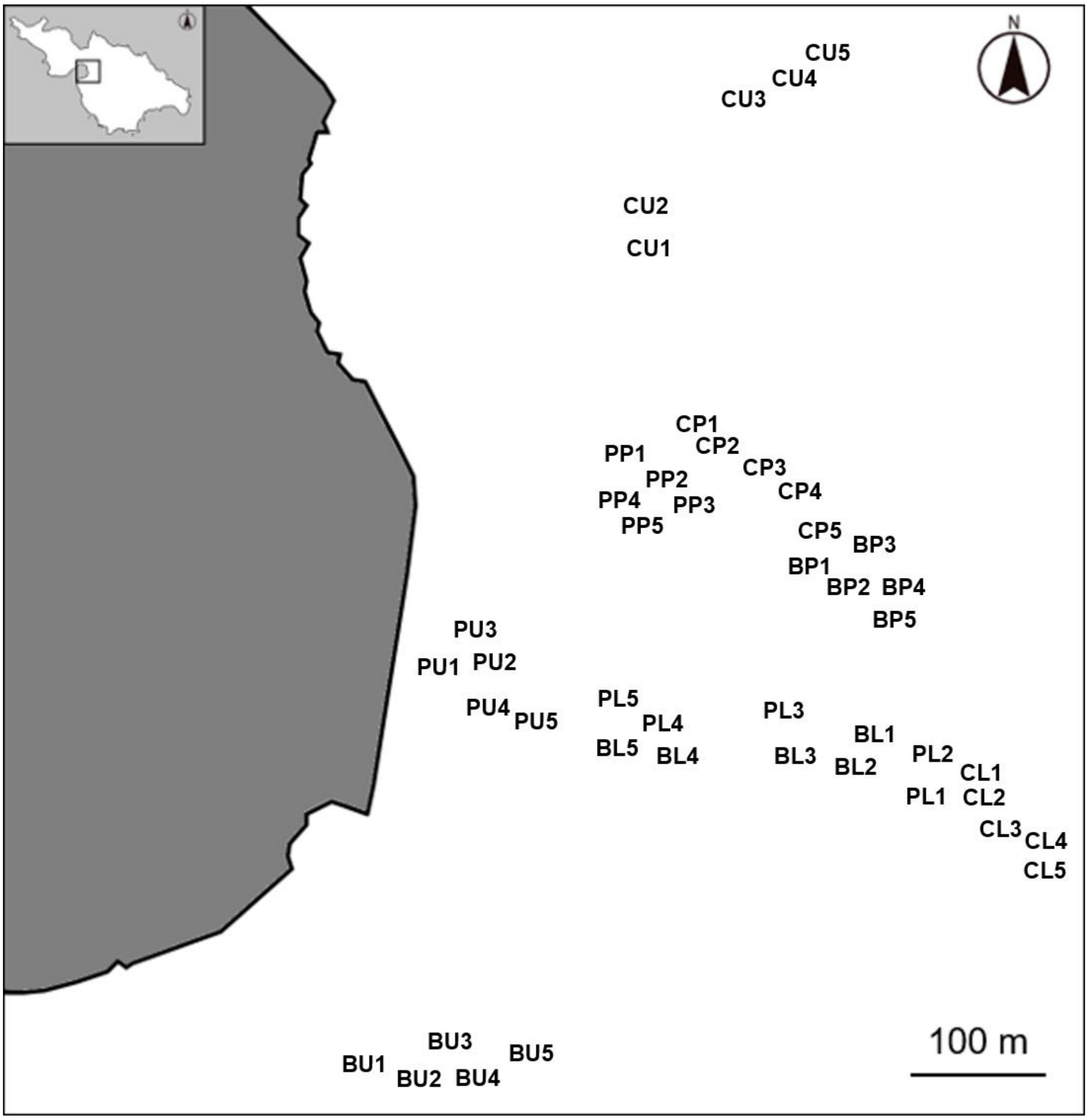

Fig. S1. Sampling points of ground-dwelling arthropods from three stand types (broad-leaved, black pine, and cedar) of each disturbance condition (control, pyroclastic flow, and lahar) following the 2015 eruption of Shin-take in Kuchinoerabu-jima, Kagoshima Prefecture, Japan. The abbreviations are as follows: broad-leaved, undisturbed forests (BU), black pine, undisturbed forests in the undisturbed area (PU), cedar, undisturbed forests (CU), broad-leaved forests with pyroclastic flow (BP), black pine forests with pyroclastic flow (PP), cedar forests with pyroclastic flow (CP), broad-leaved forests with lahar (BL), black pine forests with lahar (PL), and cedar forests with lahar (CL).

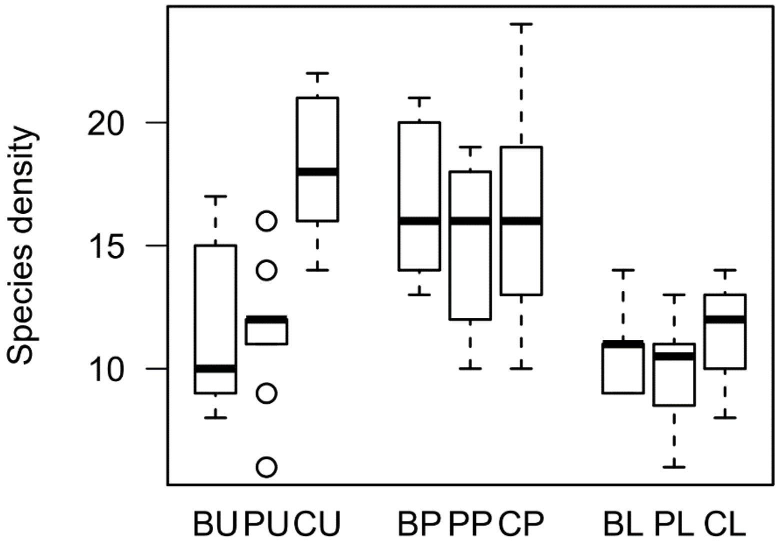

Fig. S2. Species density (non-rarefied richness) of ground-dwelling arthropods under different combinations of disturbance conditions and vegetation types. Abbreviations: BU, broad-leaved, undisturbed forests; PU, black pine, undisturbed forests in the undisturbed area; CU, cedar, undisturbed forests; BP, broad-leaved forests with pyroclastic flow; PP, black pine forests with pyroclastic flow; CP, cedar forests with pyroclastic flow; BL, broad-leaved forests with lahar; PL, black pine forests with lahar; and CL, cedar forests with lahar.

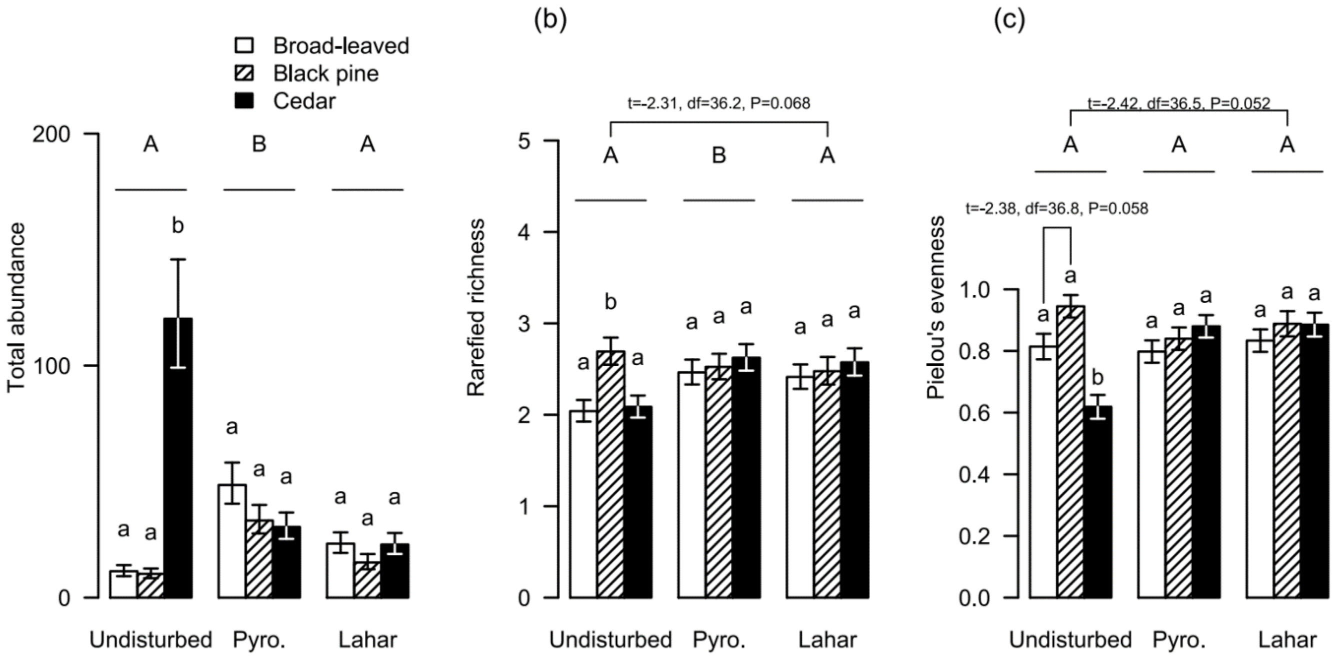

Fig. S3. Results of ant removal analyses. Differences in the (a) total abundance, (b) rarefied richness, and (c) Pielou's evenness of ground-dwelling arthropods among the three vegetation types (broad-leaved forest, black pine forest, and cedar forest) under each disturbance condition (undisturbed, pyroclastic flow, and lahar) following the 2015 eruption of Shin-take, Kuchinoerabu-jima. Back-transformed least square means ± SE are shown. Different uppercase and lowercase letters indicate statistical significance among the disturbance conditions and the vegetation types within a disturbance condition, respectively.

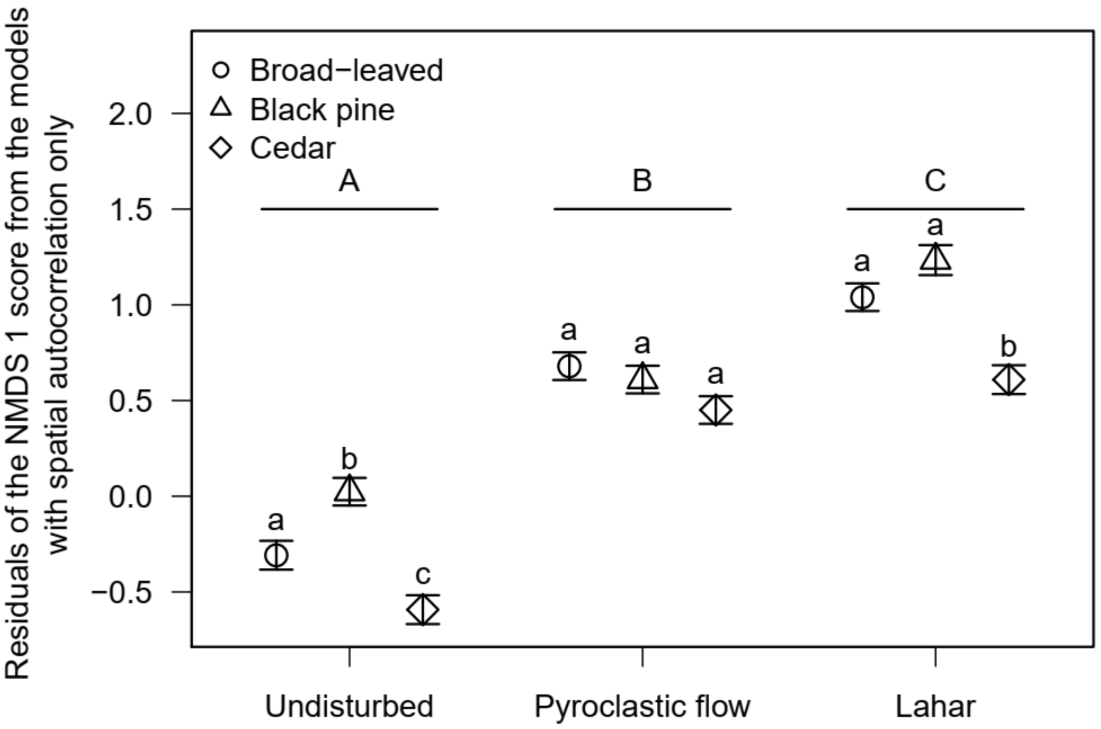

Fig. S4. Differences in the arthropod species composition after removing potential spatial autocorrelations (using the residuals from the linear model that contained the values of NMDS axis 1 as the response variable but did not contain explanatory variables other than the intercept and assumed spherical spatial correlation) among the three vegetation types (broad-leaved forest, black pine forest, and cedar forest) under each disturbance condition (undisturbed, pyroclastic flow, and lahar) following the 2015 eruption of Shin-take in Kuchinoerabu-jima. Back-transformed least square means ± SE are shown. Different uppercase and lowercase letters indicate statistical significance among disturbance conditions and vegetation types within a disturbance condition, respectively.

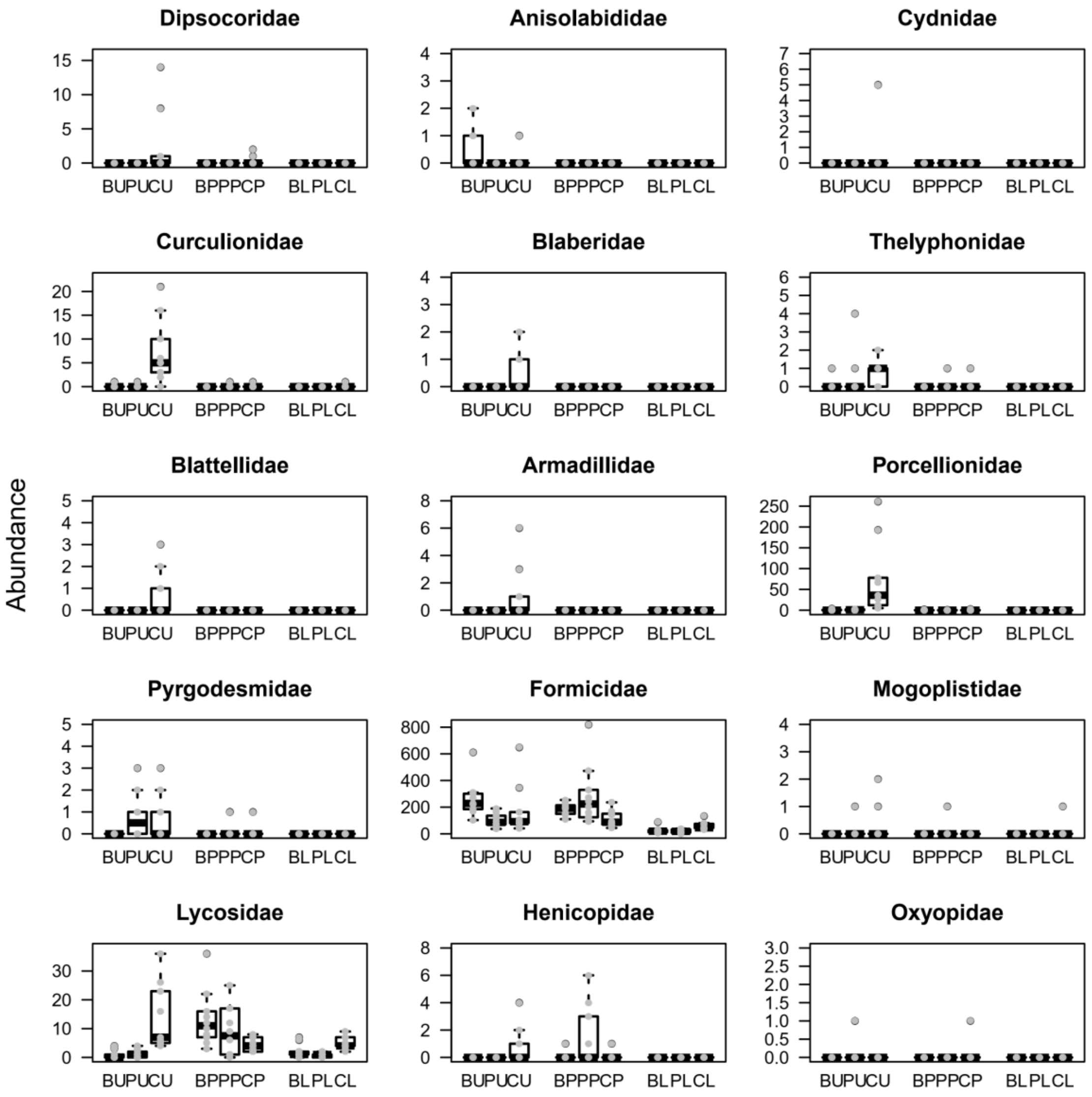

Fig. S5. Box plots of the 15 families for which the abundance significantly decreased along the NMDS axis 1. Grey points represent raw data. Abbreviations: BU, broad-leaved, undisturbed forests; PU, black pine, undisturbed forests in the undisturbed area; CU, cedar, undisturbed forests; BP, broad-leaved forests with pyroclastic flow; PP, black pine forests with pyroclastic flow; CP, cedar forests with pyroclastic flow; BL, broad-leaved forests with lahar; PL, black pine forests with lahar; and CL, cedar forests with lahar.

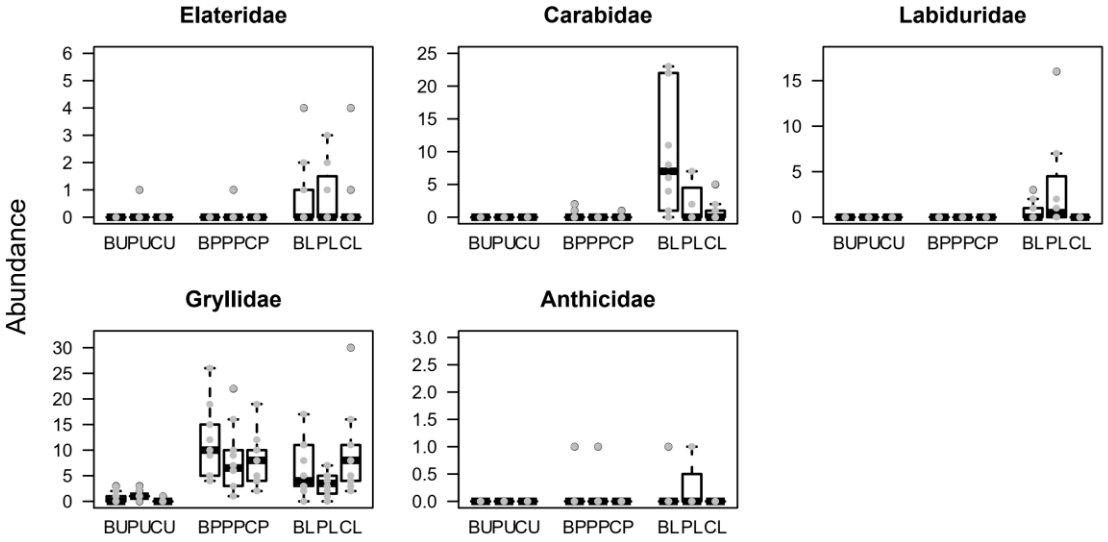

Fig. S6. Box plots of the 5 families for which the abundance significantly increased along the NMDS axis 1. Grey points represent raw data. Abbreviations: BU, broad-leaved, undisturbed forests; PU, black pine, undisturbed forests in the undisturbed area; CU, cedar, undisturbed forests; BP, broad-leaved forests with pyroclastic flow; PP, black pine forests with pyroclastic flow; CP, cedar forests with pyroclastic flow; BL, broad-leaved forests with lahar; PL, black pine forests with lahar; and CL, cedar forests with lahar.

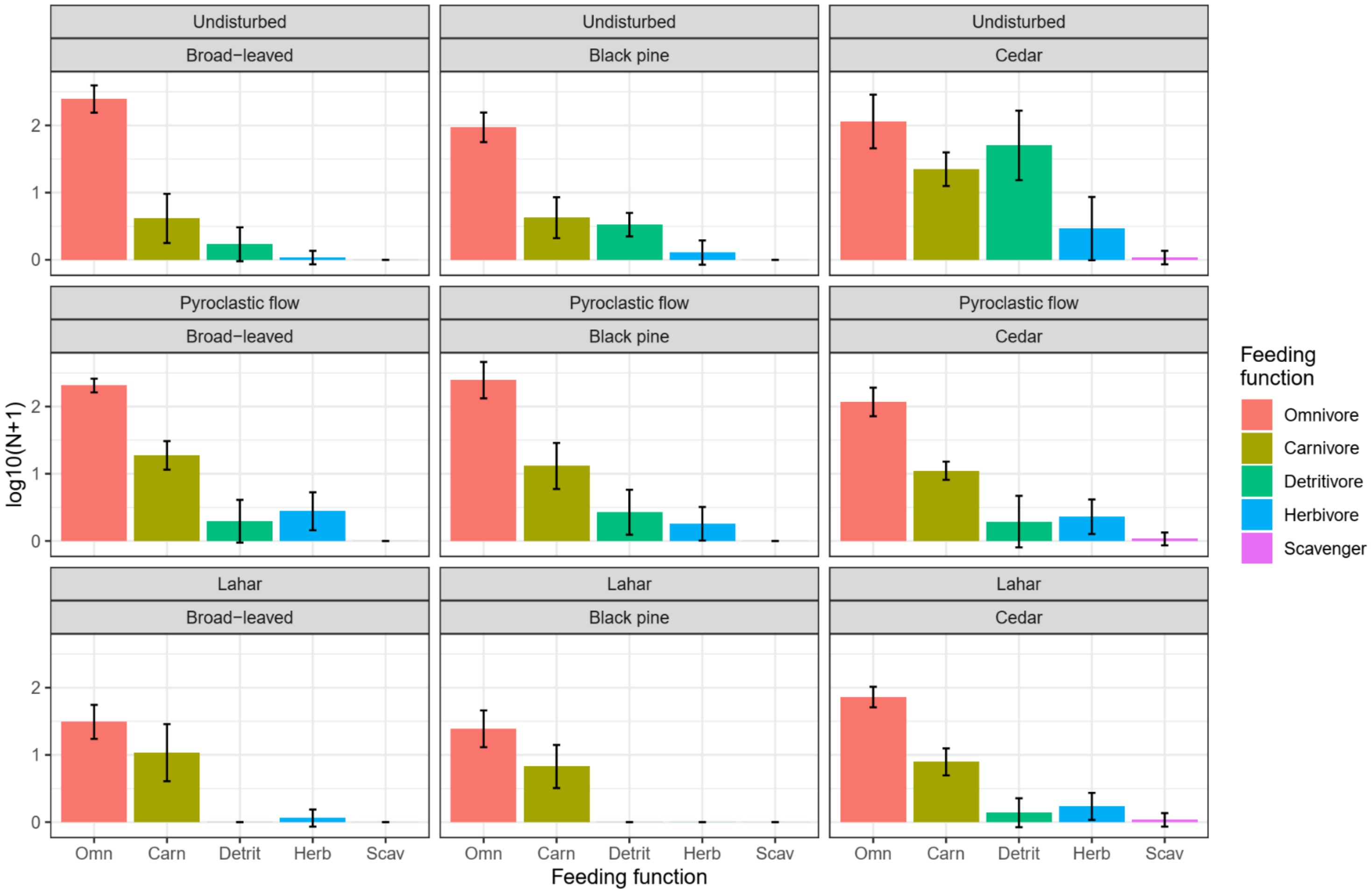

Fig. S7. The composition of the feeding functions (i.e., omnivores, carnivores, detritivores, herbivores, and scavengers) of ground-dwelling arthropods under different combinations of disturbance conditions and vegetation types. The log10-values of the number of individuals are shown (mean ± SD).
